# Task-Shifting and Quality of HIV Testing Services: Experiences from a National Reference Hospital in Zambia

**DOI:** 10.1371/journal.pone.0143075

**Published:** 2015-11-25

**Authors:** Sheila Mwangala, Karen M. Moland, Hope C. Nkamba, Kunda G. Musonda, Mwaka Monze, Katoba K. Musukwa, Knut Fylkesnes

**Affiliations:** 1 Virology Laboratory, Department of Pathology and Microbiology, University Teaching Hospital, Lusaka, Zambia; 2 Centre for International Health, Department of Global Public Health and Primary Care, University of Bergen, Bergen, Norway; 3 Pathogen Molecular Biology Department, London School of Hygiene and Tropical Medicine, University of London, London, United Kingdom; 4 Department of Public Health, School of Medicine, University of Zambia, Lusaka, Zambia; Rutgers University, UNITED STATES

## Abstract

**Background:**

With new testing technologies, task-shifting and rapid scale-up of HIV testing services in high HIV prevalence countries, assuring quality of HIV testing is paramount. This study aimed to explore various cadres of providers’ experiences in providing HIV testing services and their understanding of elements that impact on quality of service in Zambia.

**Methods:**

Sixteen in-depth interviews and two focus group discussions were conducted with HIV testing service providers including lay counselors, nurses and laboratory personnel at purposively selected HIV testing sites at a national reference hospital in Lusaka. Qualitative content analysis was adopted for data analysis.

**Results:**

Lay counselors and nurses reported confidentiality and privacy to be greatly compromised due to limited space in both in- and out-patient settings. Difficulties in upholding consent were reported in provider-initiated testing in in-patient settings. The providers identified non-adherence to testing procedures, high workload and inadequate training and supervision as key elements impacting on quality of testing. Difficulties related to testing varied by sub-groups of providers: lay counselors, in finger pricking and obtaining adequate volumes of specimen; non-laboratory providers in general, in interpreting invalid, false-negative and false-positive results. The providers had been participating in a recently established national HIV quality assurance program, i.e. proficiency testing, but rarely received site supervisory visits.

**Conclusion:**

Task-shifting coupled with policy shifts in service provision has seriously challenged HIV testing quality, protection of confidentiality and the process of informed consent. Ways to better protect confidentiality and informed consent need careful attention. Training, supervision and quality assurance need strengthening tailored to the needs of the different cadres of providers.

## Background

HIV testing and counseling (HTC) remains a critical entry point to prevention, treatment and care. Since the mid-1980s, HIV testing has been offered mainly through client-initiated voluntary counseling and testing (VCT) [1,2]. VCT is offered widely and mainly through health facilities, stand-alone facilities and mobile services such as home-based and other community-based settings [3,4,5,6]. Uptake has been disappointingly low despite high willingness to be tested, a contrast indicating acceptability barriers to the way VCT is usually offered [7,8]. As antiretroviral treatment (ART) became more available, Provider Initiated Testing and Counseling (PITC) or Routine opt-out Testing and Counseling (RTC) has been recommended to be implemented in countries with generalized epidemics with the aim to increase test rates. Evidence of increased test rates has been documented [9,10,11].

Due to the increasing need for HIV testing, testing technologies have shifted from complex techniques performed by highly trained laboratory professionals, to rapid tests (RTs) or point-of-care (POC) tests using the finger-prick method [12,13]. RTs are relatively easy to perform, are accurate and can provide same-day results and post-test counseling in a single visit [14,15,16,17]. Accordingly, RTs have paved the way for task-shifting, i.e. a process of delegating tasks from more specialized to less specialized health workers, by including non-laboratory personnel such as lay counselors and nurses to perform tests in addition to counseling [12,13,14,18,19]. Task-shifting has been one of several possible solutions to the grave human resource shortage facing the African health sector [20]. However, this expansion might affect quality of testing in various ways [12]. Quality testing demands accurate, reliable and timely test results and confidence conferred in the clients is a cornerstone [21]. An HIV test result has a huge bearing on treatment and prevention decisions, not to mention psychological effect on individuals. Given the high volume of testing, even a small error rate can result in a high number of misdiagnosed cases, emphasizing the need for quality assurance systems to regulate and monitor performance in HIV testing [12,13,22].

The provider-client relationship is a critical component in HIV testing services. While standard protocols outline appropriate information that accompanies testing, it is the counseling skills that impact on the client’s experience. Assurance of quality of counseling is important for ensuring that human rights are respected and the clients’ needs are met [23]. However as testing models such as PITC have been scaled-up, concerns have been raised on a diminished focus on preventive counseling and the consent process, leaving clients with the perception of HIV testing to be mandatory [24,25,26]. Several studies note that counseling models must be flexible taking into consideration the cultural context of family care and consultation [27,28,29]. Clients have been concerned with being seen entering or exiting an HIV testing facility, underscoring the importance of privacy and confidentiality [30,31,32,33]. In addition, building trust and rapport and imparting a non-judgmental attitude towards clients have been identified as central [32]. Other studies emphasize the need for ongoing support and supervision of providers, particularly concerning their own fears of HIV infection and difficulties in talking to clients about sexuality and disclosure of results [34,35]. Providers serve multiple roles including support, guidance, education, provision of referrals to treatment and other services and this suggests the importance of a multifaceted training [32,33,34,36]. On the whole, adherence to five key principles, the 5C’s, i.e. consent, confidentiality, counseling, correct results and connection to prevention, treatment and care is critical for any successful HIV testing service [37].

Zambia has been severely affected by the epidemic, with an adult HIV prevalence of 13.5% [38]. From 1998, Zambia rapidly expanded VCT services which are available country-wide [39]. The government has scaled-up PITC mainly through Prevention of Mother to Child Transmission (PMTCT) programs and increasingly in general health services [40]. As in other African countries, one of the main obstacles to expanded HIV testing services has been shortage of human resources. With the advent of RTs, the Ministry of Health (MOH) introduced task-shifting of HIV testing services systematically among health providers of various backgrounds and eventually to include non-health care providers, the lay counselors [41,42,43,44]. A three-day national HIV rapid testing training based on a curriculum adapted by the MOH is provided to prospective providers [45]. Despite the rapid task-shifting and scale-up of HIV testing services, studies that have described providers’ roles and experiences in providing HIV testing services in high HIV prevalence countries are few. In this study our aim was to explore various groups of providers’ experiences in providing HIV testing services and their understanding of elements that impact on quality of service.

## Methods

### Study area

The study was conducted in 2013 at the University Teaching Hospital (UTH), a third (III) level hospital with a bed capacity of 1800 and an immediate and national catchment population of 2 million and 13 million respectively. The hospital serves as the country’s specialist centre and is the main medical training institution for health professionals. UTH provides a full range of primary, secondary and tertiary health care services to both in- and out-patients and a wide range of diagnostic testing services. HIV testing is offered in form of VCT, PITC and outreach services. There are 33 HIV testing sites at the hospital [46].

### Data collection and analysis

The study was conducted at 6 purposively selected HIV testing sites at the UTH namely Adult Filter clinic, Chest clinic, Family Support Unit and the laboratories including the Virology laboratory (national reference laboratory (NRL) for HIV testing), the Sexually Transmitted Infections laboratory, and the Pediatrics laboratory. The selection was based on type of service provided, adequate client/patient flow and testing volume. Purposive sampling technique which employed a maximum variation sampling strategy was used to select study participants. With the assistance of the test site in-charges, a total of 16 providers of various ages, professional background and work experience were recruited to participate in the study. The study participants are described in Table 1. Recruitment of study participants was ended after we got a sense of saturation through observing major recurrent patterns of experiences in HIV testing service provision in the data material.

**Table 1:** Demographic characteristics of the providers.

Characteristic	N
**Profession**	
Lay counselors	4
Nurses	4
Laboratory scientists	4
Laboratory technologists	4
**Sex**	
Male	8
Female	8
**Age (yrs)**	
20–40	10
21–64	6
**HIV testing training**	
HIV rapid testing	12
Psychosocial	1
HIV rapid testing and psychosocial	6
No formal training	3
**HIV testing work experience**	
≤ five years	8
6–20 years	4
Over 20 years	4

A total of 16 in-depth interviews (IDIs) and 2 focus group discussions (FGDs) were conducted with the providers. IDIs explored individual experiences, while FGDs aimed to add and validate the findings from the IDIs. The IDIs were guided by a semi-structured interview guide drafted prior to the study. The interview guide consisted of open and closed-ended questions and described main topics as follows: demographic characteristics, HIV testing training and experience, workload, test kit management, counseling experience, privacy, confidentiality and consent and service infrastructure. Without changing the questions or content, the interview guide was revised and further developed throughout the interview process by adjusting the probes for some questions in order to have more in-depth responses. The FGDs were guided by a topic guide covering the same topics as the interview guide. As the FGDs were conducted at the end of the data collection, they incorporated information obtained from the IDIs. The interviews were carried out in a private room at the sites or home, depending on the convenience and preference of the informants. The FGDs were conducted in a separate room provided at the institution which offered an adequate, quiet and private environment. Each group comprised of 8 participants, of which 2 participants were from each provider group. The reason for having a mix of professions was to have a more diverse discussion on issues regarding quality service provision. We experienced a high level of participation and a rich discussion. The FGDs provided a platform for both learning and sharing knowledge between the different provider groups. The interviews were conducted by the first author (a Zambian biomedical scientist with epidemiology specialization), with the assistance of an experienced Zambian biomedical and public health scientist with interviewing skills and experience in qualitative work. The FGDs were conducted by trained public health scientists, while the first author observed group dynamics and took notes. The IDIs and FGDs were conducted mainly in English, with Bemba and Nyanja (local languages) words occasionally employed where necessary. Each IDI took about 1½ hours, while the FGDs took about 2 hours. The IDIs and FGDs were audio-recorded, transcribed verbatim and translated by the first author and the assistant. The aim was to retain the transcripts verbatim and translations as close as possible to the actual stated content. The early interviews were followed by a thorough discussion of the content with the research team, as part of continuous reflection and analysis.

The analysis was performed by drawing upon qualitative content analysis [47]. The analysis firstly involved reading and re-reading of the field notes and transcripts, as well as listening to the audio recordings several times for familiarization. The content was carefully coded manually in the margins. The data was then synthesized and grouped into relatively exhaustive categories, and from which central themes were identified. Comparisons were made continuously both within and between the transcripts to reveal commonalities and patterns, as well as contradictions in the experiences of HIV testing service provision. As the IDIs and FGDs covered the same topics, it was deemed appropriate to integrate both in the analysis. Prior to the analysis, a preliminary list of codes was developed based on the interview guide topics. Subsequently, the coding list was revised and expanded inductively based on key issues that emerged from reading the transcripts. To validate the coding process two authors independently coded two interviews and discrepancies were resolved. To ensure reliability of the coding system, authors communicated regularly to integrate changes in the codes.

### Ethical clearance

Ethical clearance was obtained from the University of Zambia Biomedical Research Ethics Committee (UNZABREC) and the University of Bergen Research Ethics Committee (UibREC). In addition, permission was obtained from the UTH authority and the site in-charges to conduct the research at the sites. Participation was voluntary and all the participants provided written informed consent for participating in the study. No participant withdrew or refused to participate. Confidentiality and anonymity of the informants were emphasized and maintained throughout the study.

## Results

### Demographic characteristics of the providers

The providers were lay counselors (4), nurses (4), laboratory scientists (4) and laboratory technologists (4) (Table 1). Two nurses were in-charges while three laboratory scientists were national HIV testing trainers. Of the 16 providers, 8 were male and 8 were female. The age range of the informants was from 20 to 64 years, of which nearly two-thirds were aged between 20 and 40 years. The majority (12) reported to have received the standard national HIV rapid testing training and of which half (lay counselors and nurses) had also received the psychosocial counseling training. A few providers (4), particularly laboratory personnel, had no formal HIV testing training, but reported to have learnt to perform testing from the supervisor or colleagues. In this setting, laboratory scientists, who hold degrees in Biomedical or Biological Sciences, have higher education levels than laboratory technologists who hold diplomas in Biomedical Sciences. Both professions however, undergo the same HIV rapid testing training and perform the same testing. Half the providers had less than 5 years HIV testing experience while four had been testing for over 20 years. All lay counselors and nurses reported to provide both VCT and PITC services. The laboratory providers tested samples for several purposes including VCT, PITC and routine medical examinations.

The providers’ experiences of HIV testing service provision and their understanding of elements impacting on quality were grouped under four main themes: Threats to confidentiality and informed consent; Non-adherence to testing procedures and implications for quality care; High workload and stress; Inadequate training and quality assurance. Table 2 provides a detailed presentation of the description of each element subsumed under each theme.

**Table 2:** Providers’ experiences in HIV testing service provision and their understanding of elements impacting on quality.

Theme	Service element description	Provider group
Threats to confidentiality and informed consent	Confidentiality difficult to maintain due to limited space in both in-patient and out-patient settings	Lay counselors; Nurses
	Difficulties in upholding consent in PITC: Patients not willing to be tested, application of persuasion to consent to testing; reduced pre-test counseling	Lay counselors; Nurses
Non-adherence to testing procedures and implications for quality care	Difficulties in finger pricking and obtaining adequate blood volumes for testing	Lay counselors
	Using more than recommended volume of buffer to quicken test procedure	Lay counselors
	Substituting buffer with normal saline or other test kits buffers due to non-availability of buffer	All providers
	Giving positive result based only on the screening test due to non-availability of confirmatory tests	Laboratory personnel (with limited HIV testing training)
	Rushing to report results before test set time and possibility of giving inaccurate results	All providers
	Difficulties describing, interpreting and understanding causes of false-negative, false-positive and invalid results	Lay counselors; Nurses
High workload and stress	High workload leading to rushed testing and counseling	Lay counselors (primarily)
	High emotional stress due to dealing continuously with difficult patient situations e.g. giving positive results, rape victims.	Lay counselors
Inadequate training and quality assurance	Training duration too short, more practical sessions needed especially in finger pricking.	Lay counselors
	No refresher trainings. Refresher training perceived important as a ‘revision’ or ‘reminder’.	All providers
	No supervisory visit by trainers after training. Supervisory visit perceived important for assessing competence and for moral support.	All providers
	IQC not performed consistently. EQA conducted once yearly in form of PT, but SSV rare.	All providers

### Threats to confidentiality and informed consent

Ensuring confidentiality and privacy and upholding the principle of consent were two elements that emerged as critical and having an impact on quality of service.

#### Confidentiality is not there

Lay counselors and nurses reported that though maintenance of confidentiality and privacy were critical to the development of the test counseling relationship, to client trust and disclosure, they were difficult to maintain due to limited space: *“Confidentiality is not there*. *Sometimes you (providers) are more than one in a room*. *Sometimes you have students in the same room*. *I think clients don’t feel at ease*, *they can’t say everything like they wanted*, *it’s supposed to be a one to one thing*. *The room is too small*.*”* (Lay counselor, IDI). Another lay counselor said: “*It’s also very difficult for me to say everything properly*. *And imagine a client’s result comes out positive; that is a blow for our clients”*. Some providers also felt that for this reason they tend to lose clients: *“…some of them (clients) just say ‘naaya’ (meaning I’m going)*.*”* (Lay counselor, IDI). A few providers reported that before the start of the session the client would be asked if they were comfortable having other staff or students present in the session and that if they objected, the staff or students would be asked to go out. However, other providers felt that some clients would not complain due to them not being aware of their right to privacy, or they were too scared to say: “*I think it’s very rare for clients to complain because some don’t know how it is supposed to be done*, *some are intimidated they wouldn’t just open up*.*”*(Nurse, IDI). Field observations noted that the sharing of limited space by the staff and students led to interruptions during sessions with people going in and out. The providers also reported that previously some clients had expressed concern about being seen entering the VCT room by someone they knew and this led to a change in system, where VCT labels were removed from the doors and all patients had one common waiting area.

Regarding PITC in the wards, most of the lay counselors and nurses felt that it was difficult to maintain confidentiality and privacy in this setting: *“In the wards*, *we do diagnostic testing*, *we test each and every patient*, *there is no privacy*, *beds are so close together*, *some patients on the floor*, *sometimes there are no curtains to close and relatives are present*, *you have to move very close to the patient when communicating so that other people don’t listen to the conversation*, *which again is not healthy*.*”* (Lay counselor, IDI). Other providers reported that in situations where the patient was too ill, communication was made with the bed-sider, i.e. family member or relative.

#### Persuasion for consent

Most lay counselors expressed challenge in implementing PITC, to make patients understand why an HIV test had to be done, as most patients were not willing to be tested: *“…most patients are not willing to do the test because they have come for something else*. *Here we say ‘we have to do the test’ and they say*, *‘No I just came for headache and now you are saying I should do an HIV test’*, *it’s difficult*.” (Lay counselor, IDI). The providers noted that in such situations more time was given to the patient, or they simply accepted the patients’ choice. However the providers described situations where some persuasion was applied for the client to understand and take the test, which some providers during the FGD felt countered HTC guidelines for eliciting voluntary informed consent. The providers also reported difficulties in counseling patients who were very ill: *“…sometimes counseling a patient who is not feeling well is difficult*, *you are talking*, *the patient is not responding well*.*”* (Lay counselor, IDI). Counseling was noted to be limited in the ward setting; one lay counselor said *“…it’s not necessary to tire them with all the explanations*, *you just tell them what is necessary and how it (HIV) is probably connected to what they are suffering from*.*”*

### Non-adherence to testing procedures and implications for quality care

The importance of adherence to testing procedures and implications of non-adherence emerged as a central theme in our interviews and discussions. Most providers reported that non-adherence to the testing guidelines could lead to inaccurate results: *“If one does not stick to the right procedure*, *you’ll end up giving an inaccurate result*. *One should not depart from laid down procedures at any time*.*”* (Lay counselor, FGD).

#### Missing skills

Though most providers reported that they found performing an HIV rapid test to be easy, some providers particularly lay counselors reported to have difficulties in finger pricking and blood drawing: *“…sometimes it’s difficult to get the volume of blood required for the test*, *especially for anemic patients… but I just use what I get for the test…”* (Lay counselor, FGD). Some providers and trainers reported that finger pricking and drawing adequate blood volumes could be difficult in situations such as cold weather, callused patient fingers or wrong type of lancet and that it required adequate training and practice to perfect the skill. The trainers reported that using inadequate blood volumes for the test could lead to inaccurate results: *“…with little blood*, *providers tend to use more buffer (chemical that assists the lateral flow of the specimen) thereby diluting the blood and reducing the antibody titres to be detected by the test and could lead to false-negative results*.*”* (Laboratory scientist, trainer, FGD).

#### Missing tools

Some of the providers reported to sometimes fail to adhere to certain procedures, partly due to non-availability of reagents such as the buffer and having to substitute with other test kit buffers or normal saline: *“I have done that before yes*, *I just used that because it was a buffer*, *no serious problem about it*.*”* (Laboratory technologist, IDI). Another reason reported by lay counselors was to quicken the test procedure e.g. by adding more than recommended volume of buffer without understanding the implications of such actions.

Non-availability of test kits, particularly confirmatory test kits due to delays or inadequate supplies from the national distributor, Medical Stores Limited (MSL), was seen to lead to non-adherence to the testing algorithm, which specifies the use of two rapid blood antibody assays i.e. Determine^®^ HIV-1/2 (Abbott Laboratories, Abbott Park, IL) as a screening test and if reactive, Uni-Gold^TM^ HIV (Trinity Biotech Plc, Wicklow, Ireland) as a confirmatory test. This was observed through a few laboratory personnel with limited HIV testing training who reported to have been issuing a positive result based on the screening test only. Some providers and trainers expressed the danger of reporting positive results based on the screening test only, which was highly sensitive, and the possibility of issuing false-positive results and stressed the importance of confirmatory testing which was highly specific, to rule out false-positive results: *“Sometimes when someone is carrying antibodies to other diseases that are mimicking those of HIV*, *they are picked up by the ‘determine’*, *so if you are not confirming the ‘determine’ results you can give someone a false-positive result*.*”* (Laboratory technologist, IDI). All lay counselors, nurses and most laboratory personnel however reported that in cases of stock-outs of the confirmatory kits, the testing service was not offered as per the guidelines which specified the use of both kits.

#### Rushing to give the result

Some providers and trainers reported that correct timing of the test procedure was an important factor and that rushing to give a result, particularly a negative result, could lead to giving a false-negative result: *“I know a colleague who rushed in reporting the result as negative and after sometime we saw it was positive*. *Unfortunately the patient had already received their results*, *but they hadn’t left yet*. *So we told the counselors who then explained to the patient what had happened and gave them the correct result*.*”* (Laboratory technologist, IDI).

Handling of false-negative results was reported to be a dilemma for some providers. On the one hand it was important to inform the client immediately in order to reduce risk, but on the other hand admitting that the result was wrong was a burden to the provider and could compromise trust in the service: *“It’s difficult if you have already told them they are negative and then you call them back to tell them they are positive*, *no*. *Since you have already advised them to come back and test after window period (3 months)*, *when they come then you repeat the test*. *They may say you are playing with people’s lives or think you are lying*.*”* (Lay counselor, IDI). Invalid results were reported to be uncommon, but were usually due to use of inadequate volume of specimen or defective test devices. Some providers, particularly lay counselors and nurses, however, reported not to have come across false-positive, false-negative and invalid results and had some difficulties describing, interpreting and explaining the possible causes of such results.

### High workload and stress

High workload was reported to have a significant negative impact on quality of service. Some of the lay counselors felt that high workload could lead to non-adherence to procedures (such as reading test results before the set time, rushed counseling), in order to clear off the workload quickly. Some expressed that in their setting, numbers (statistics) were important but could be compromising the quality of service: *“We attend to patients for PITC as well*, *in the wards and OPD*, *statistics there matter*, *the numbers shouldn’t go too down*, *so this patient comes*, *you do the test*, *other patients are waiting*. *The workload is heavy*, *today is better*, *if you came another day you would see how we do it*, *it’s terrible*. *Sometimes we even counsel two people at a go because the work is too much…you can counsel and test 30 patients in a day*.*”* (Lay counselor, IDI). High workload and stress was primarily a problem for the lay counselors who apart from performing the majority of testing also provide counseling.

Some providers, particularly lay counselors felt that their job, though rewarding was difficult and mentally tiring, exacting an emotional toll: *“Depending on the issue the client is presenting*, *some issues are difficult e*.*g*. *counseling rape victims*, *molested children*, *giving a positive result*, *some will start breaking down*, *and you are there as human as you are*, *you also get touched*. *It’s a challenge if they come so often*, *you easily get stressed out*.*”* (Lay counselor, IDI).

### Inadequate training and quality assurance

#### The need for re-training and follow-up

HIV rapid testing training was regarded as essential for quality testing. Upon training, most providers felt confident in doing their work: *“After training I was able to handle the test confidently and accurately*. *Prior to that I was just testing by looking at the manual and SOPs but after I was trained all the areas where I would go wrong were corrected*.*”* (Laboratory scientist, IDI).

Some providers, particularly lay counselors, felt that the 3-day standard national HIV testing training was too short and needed more time for practical sessions: *“The 3 days is not enough*, *the course should be extended so that you can have more practice and know well how to prick*, *which finger to prick because in some clinics where you go for practical sessions you are not allowed to prick any patients…”* (Lay counselor, IDI). This was supported by the trainers who felt that lay counselors who did not have a scientific background required more time for training: *“…lay counselors have problems understanding some of the technical or scientific things which they have to learn in a short time*. *But for someone with a scientific background 3 days is enough as they understand some things*. *Adding a few more days would be much better*.*”* (Laboratory scientist, trainer, IDI).

Refresher training, which most providers reported to have never received from the time they had their initial training, was seen to be important as a ‘revision’ or ‘reminder’, as well as a way of learning new methods in HIV testing which could further improve quality of testing. This was supported by the trainers who also felt that refresher trainings were important to ensure that providers were conducting testing in a standard way: *“Refresher trainings are important because when providers go out there they tend to relax with time and have shortcuts which lead them to make mistakes when testing*. *So if once in a while we do refresher training*, *the providers will be moving at the same pace*.*”* (Laboratory scientist, trainer, IDI).

Both the trainers and providers felt that a follow-up visit by the trainer after training was important to give the trainer an opportunity to assess the performance of the providers in their respective work stations and to provide guidance: *“The most important thing in training is a follow-up visit by the trainer to see what challenges the providers will face in the field because some of those challenges cannot come up in a short period of 4 hours (training practical session)*, *but in a longer period of time*.*”* (Laboratory scientist, trainer, FGD). Most providers reported to have never had any follow-up visit from their trainer.

#### Participation in quality assurance programs

Though the providers reported to be carrying out internal quality control (IQC), i.e. a measure to ensure test precision is optimal, most and particularly non-laboratory providers reported that it was not being done consistently as per guidelines. All providers reported to be participating in the external quality assessment (EQA) program conducted by the Zambia National Quality Assurance Program (ZANQAP) of the NRL. The main EQA activities reported included annual proficiency testing (PT), i.e. a system where simulated specimens are issued to testing sites by the NRL and performance is assessed by comparison of reported results to the expected results, and site supervisory visits (SSVs), i.e. a system that involves an on-site review of all aspects of a quality system, which were rare. Most providers expressed the importance of such activities to monitor their performance in HIV testing, but suggested more frequency to ensure proficiency: *“I feel quality assurance checks and visits should be stepped up*, *be on a regular basis*, *you know we are only humans*, *to ensure that there is no messing up with the systems*, *or compromising the algorithm…”* (Lay counselor, IDI).

## Discussion

Task-shifting and shifts in service provision policies have affected quality of service. Lay counselors and nurses reported limited space to greatly compromise privacy in both in- and out-patient settings. Grave difficulties in upholding consent were reported in provider-initiated testing in in-patient settings. The providers identified non-adherence to testing procedures, high workload and inadequacies in training and supervision as key elements affecting quality of testing. Difficulties in testing varied by sub-groups of providers: lay counselors, in finger pricking and obtaining adequate specimen volumes; non-laboratory staff, in understanding and interpreting invalid and false results. The providers reported participating in national quality assurance programs, i.e. proficiency testing, but seldom received site supervisory visits.

PITC as part of all out-patient health services has been extensively scaled up in antenatal care and been increasingly implemented in general health services in Zambia [40]. Our findings indicate that providers face a number of challenges in implementing PITC, particularly in in-patient settings. Firstly, the finding of patients not willing to be tested and the application of persuasion for clients to consent to testing was of concern. The implementation of the PITC model was perceived to be mandatory as reflected in the formulation ‘we have to do the test’ by the providers, which runs counter to current guidelines which emphasizes clients’ rights to opt-out of HIV testing [9]. A study in Botswana showed that 68% of the participants felt that they could not refuse the test [48]. Secondly, the finding of limited pre-test counseling and replacement with ‘simplified pre-test information’ is consistent with other publications that have raised the issue of neglected counseling in this testing model [4,24,25]. Pre-test counseling plays a fundamental role; entirely removing it or providing inadequate information reduces the opportunities for eliciting informed consent and potentially makes receiving a positive test result more difficult to handle [49,50]. Thirdly, the closeness of patients’ beds, the presence of relatives by the bed-side and the relaying of information to the bed-sider in situations where the patient was too ill led to inadvertent compromises in patient confidentiality. Breaches in confidentiality and privacy were also reported in out-patient settings due to limited space. The WHO/UNAIDS guidelines state the need to protect confidentiality of the patient test result, without the engagement of a third person or relatives they have chosen not to bring into the process [9]. The findings indicate that the process of translating PITC policy into practice is complex and further in-depth research is needed to explore challenges faced by providers and how to strengthen health systems, particularly in in-patient settings [51]. The need for scale-up of HIV testing is beyond doubt. However, much attention is needed not to erode ethical and human rights obligations [52]. It is important to be cognizant of what is lost in the process if PITC is not implemented with caution and with respect for the principles on which it is based [26].

Non-adherence to testing procedures was seen as an important aspect impacting on accuracy of test results, confirming previous study findings [53]. The use of an inadequate volume of specimen for testing, or the addition of more than recommended volumes of buffer to quicken the test procedure has been reported to lead to suboptimal test performance with poor sensitivity or specificity [13]. Rapid tests have sometimes been found to have low sensitivity particularly during sero-conversion; therefore reading results before the set time could lead to false-negative results, particularly for weakly positive samples [54,55]. Though there are no documentations in literature stating that substituting a dedicated buffer of one HIV test kit with a buffer of another HIV test kit brand or normal saline could lead to inaccurate results, such practices have been found to lead to inaccurate results for other diagnostic tests, i.e. a study in Belgium found that buffer substitution in malaria rapid diagnostic tests leads to false-positive results [56]. The issuing of positive test results based on the screening test only raised a serious concern about some of the providers’ level of understanding. Accuracy in HIV rapid testing cannot be overemphasized; false-negative or false-positive results have direct implications for individuals for treatment, prevention and transmission [13].

Workload was reported to be high among most providers and particularly among lay counselors, who apart from testing also provide counseling in both VCT and PITC settings. High workload was reported to impede quality of service, confirming previous publications that have found this to be true, particularly in public institutions [57,58,59]. These findings suggest the need for the review of workload for all providers and particularly for lay counselors, by either increasing the number of staff to handle the high client flow or moderating the client flow. Previously lay counselors only performed counseling as testing was restricted to health professionals [60]. The finding that the bulk of testing has been done by lay counselors indicates that task-shifting in HIV testing services has taken effect in Zambia [44]. Hence there is a need to concentrate efforts to support and ensure quality service provision among these groups of providers. Further, lay counselors reported to have high emotional stress due to the highly emotional patient situations that they deal with daily. Mentorship and education on emotional labor through supportive supervision have been suggested to be helpful [36].

Training was seen as an important aspect which enabled the providers to have confidence in their work, confirming previous publications [30,31]. However, the fact that some providers were performing testing without formal HIV testing training raised a great quality concern. Previous research indicates that limited training affects accurate diagnostic testing [12,15,22]. All prospective testers should therefore be trained and certified upon a competency assessment before practicing and releasing results to clients [13]. Our finding that lay counselors and nurses were not able to clearly describe, interpret and explain the causes of a false-negative, false-positive and an invalid result raised a concern about their ability to handle difficult results. Further, the finding that lay counselors were having difficulties in finger pricking and needing more training supports previous findings [15,22]. These findings, coupled with the trainers’ reports suggest the need for training to be tailored, with particular attention to lay counselors who have more testing challenges than other cadres of providers. The informants were concerned about lack of refresher training since it was perceived to be of great importance for updating skills and maintaining competences, supporting previous study observations [13,60,61]. Further, the reported lack of supervision after training indicated the need for strengthening of resources in the training system to support trainers to visit, provide moral support and address challenges faced by their previous trainees [12,13,57].

Quality assurance systems were considered to be vital for monitoring HIV testing among testing sites. The fact that most providers were not performing IQC consistently according to guidelines raised a concern. Studies have found that non-performance of IQC could lead to non-detection of systematic errors that could occur during the test procedure and may lead to the release of erroneous results [61,62]. These findings suggest the need for strengthening of supervision and monitoring of IQC activities in all sites and particularly in non-laboratory sites. Regarding EQA, the providers participated in annual national PT exercises, but rarely received SSVs. The limited PTs and SSVs were reported to be due to lack of human and financial resources (personal communication with the ZANQAP manager). These findings suggest the need for better prioritization of resources.

In Zambia, the main groups providing HIV testing services include lay counselors, nurses and laboratory personnel, while other professions such as doctors and clinical officers perform testing at a very small scale [63]. Our focus was on the main groups which included a few laboratory personnel who had been testing before task-shifting. Problems in HIV testing have been there even before task-shifting, but confined to mainly one profession. But with the rising concerns about the effects of expansion and task-shifting of HIV testing services on testing quality [12], our assumption was that the problems could be more, considering that the providers are of diverse backgrounds. Our findings indicate that each group has specific challenges and requiring specific interventions.

Apart from recall bias, self-reporting on experiences is subject to social desirability bias which may have led to informants either over-reporting or under-reporting experiences. The informants may also have been influenced by anxiety of a face-to-face interview. Rapport was established with the informants through informal conversation and spending time to ensure that they were comfortable to engage in discussion. In the FGDs, the participants had the advantage of group dynamics and freely discussed their experiences. Responses may also have been influenced by the status of the interviewers, i.e. the first author and assistant, being biomedical scientists. However, the interviewers were considered as ‘one of them’ coming from the same umbrella institution, and the ones to help offer solutions to the challenges they were experiencing and this may have enhanced the responses. A deliberate effort was made to maximize the reporting of experiences by assuring informants of confidentiality, anonymity and the freedom not to answer questions they were not comfortable with.

A major strength of this study was the use of a combination of IDI and FGD data collection techniques. This increased the validity of the findings as the two techniques complemented each other, reducing potential weaknesses of using a single technique. The FGDs added unique information that did not appear in the IDIs. This study was conducted in the capital city of Zambia at the University Teaching Hospital (UTH), a tertiary hospital and main referral health institution in the country which has a comparatively high number of collaborating partners that support improved quality health services. Thus the transferability of the study findings to lower-level health care contexts in Zambia may be limited. However, the study included the main groups of providers in the country with experience from providing HIV testing services in various settings and to different target groups including VCT, PITC and outreach. Accordingly we consider the findings to have relevance for settings beyond the hospital level. However, further research carried out in different settings including rural sites, lower-level and private health facilities would be useful to fully capture the complexity involved.

## Conclusions

Task-shifting coupled with policy shifts in service provision have challenged quality of testing service. Ways to better protect confidentiality and informed consent need careful attention to ensure clients’ rights are respected. In this regard, the findings revealed critical challenges for providers when practicing the provider-initiated testing model, a finding that requires further investigation and particularly in in-patient settings. The study suggests an urgent need for strengthening of training, supervision and quality assurance systems tailored to the needs of the different cadres of providers and with a particular focus on lay counselors who appeared to have the greatest challenges.

## References

[1] UNAIDS. HIV Voluntary Counseling and Testing: A gateway to prevention and care UNAIDS Best Practice collection, ed. Geneva: UNAIDS; 2002.

[2] World Health Organization. Guidelines for counseling about HIV infection and disease Geneva: World Health Organization; 1990.

[3] Fylkesnes K, Siziya S. A randomized trial on acceptability of voluntary HIV counseling and testing. Trop Med Int Health. 2004; 9(5):566–72. 15117300 10.1111/j.1365-3156.2004.01231.x

[4] Obermeyer CM, Osborn M. The utilization of testing and counseling for HIV: a review of the social and behavioral evidence. Am J Public Health. 2007; 97(10):1762–74. 17761565 10.2105/AJPH.2006.096263 PMC1994175

[5] Bateganya MH, Abdulwadud OA, Kiene SM. Home-based HIV voluntary counseling and testing in developing countries. Cochrane Database Syst Rev. 2007; 4. 10.1002/14651858.CD006493.pub2 17943913

[6] Matovu JK, Kigozi G, Nalugoda F, Wabwire-Mangen F, Gray RH. The Rakai project counseling programme experience. Trop Med Int Health. 2002; 7(12):1064–7. 12460398 10.1046/j.1365-3156.2002.00964.x

[7] Babalola S. Readiness for HIV testing among young people in northern Nigeria: the roles of social norm and perceived stigma. AIDS Behav. 2007; 11(5):759–69. 17191141 10.1007/s10461-006-9189-0

[8] Fylkesnes K, Haworth A, Rosensvard C, Kwapa PM. HIV counseling and testing: overemphasizing high acceptance rates a threat to confidentiality and the right not to know. AIDS. 1999; 13(17):2469–74. 10597789 10.1097/00002030-199912030-00019

[9] WHO, UNAIDS. Guidance on provider-initiated HIV testing and counseling in health facilities Geneva: WHO; 2007.

[10] Ministry of Health. Adult and adolescent antiretroviral therapy protocols Lusaka: Ministry of Health; 2010.

[11] Wanyenze RK, Nawavvu C, Namale AS, Mayanja B, Bunnell R, Abang B, et al Acceptability of routine HIV counseling and testing and HIV seroprevalence in Ugandan hospitals. Bull World Health Organ. 2008; 86(4):302–9. 18438519 10.2471/BLT.07.042580 PMC2647415

[12] Yao K, Wafula W, Bile EC, Cheignsong R, Howard S, Demby A, et al Ensuring the quality of HIV rapid testing in resource-poor countries using a systematic approach to training. Am J Clin Pathol. 2010; 134(4):568–72. 10.1309/AJCPOPXR8MNTZ5PY 20855637

[13] Parekh BS, Kalou MB, Alemnji G, Ou CY, Gershy-Damet GM, Nkengasong JN. Scaling up HIV rapid testing in developing countries: comprehensive approach for implementing quality assurance. Am J Clin Pathol. 2010; 134(4):573–84. 10.1309/AJCPTDIMFR00IKYX 20855638

[14] Schalla WO, Hearn TL, Taylor RN, Eavenson E, Valdiserri RO, Essien JD. CDC’s model performance evaluation program: assessment of the quality of laboratory performance for HIV-1 antibody testing. Public Health Rep. 1990; 105(2):167–71. 2157234 PMC1580063

[15] Chiu YH, Ong J, Walker S, Kumalawati J, Gartinah T, McPhee DA, et al Photographed rapid HIV tests results pilot novel quality assessment and training schemes. PLOS One. 2011; 6:3. 10.1371/journal.pone.0018294 PMC3069085 21483842

[16] Koblavi-Dème S, Maurice C, Yavo D, Sibailly TS, N’guessan K, Kamelan-Tano Y, et al Sensitivity and specificity of human immunodeficiency virus rapid serologic assays and testing algorithms in an antenatal clinic in Abidjan, Ivory Coast. J Clin Microbiol. 2001; 39(5):1808–12. 11325995 10.1128/JCM.39.5.1808-1812.2001 PMC88030

[17] WHO. HIV assays: operational characteristics. Report 16. Rapid assays. Geneva: WHO; 2009. Available: http://www.who.int/diagnostics_laboratory/publications/Report16_final.pdf. Accessed 23 Mar 2015.

[18] WHO. Treat, train, retain: the AIDS and health workforce plan: report on the consultation on AIDS and human resources for health Geneva: WHO; 2006 Available: http://www.who.int/hiv/pub/meetingreports/TTRmeetingreport2.pdf. Accessed 13 Jan 2015.

[19] WHO: Task shifting: rational redistribution of tasks among workforce teams: global recommendations and guidelines Geneva: WHO; 2008 Available: http://www.who.int/healthsystems/TTR-TaskShifting.Pdf. Accessed 15 Jan 2015.

[20] Lehmann U, Van Damme W, Barten F, Sanders D. Task-shifting: the answer to the human resources crisis in Africa? Hum Resour Health. 2009; 7:49 10.1186/1478-4491-7-49 19545398 PMC2705665

[21] WHO. Guidelines for assuring the accuracy and reliability of HIV rapid testing: applying a quality system approach Geneva: WHO; 2005.

[22] Learmonth KM, McPhee DA, Jardine DK, Walker SK, Aye TT, Dax EM. Assessing proficiency of interpretation of rapid human immunodeficiency virus assays in nonlaboratory settings: ensuring quality of testing. J Clin Microbiol. 2008; 46(5):1692–7. 10.1128/JCM.01761-07 18353938 PMC2395071

[23] World Health Organization. A handbook for improving HIV testing and counseling services Field test version. Geneva: WHO; 2010.

[24] Yeatman SE. Ethical and public health considerations in HIV counseling and testing: policy implications. Stud Fam Plann. 2007; 38(4):271–8. 18284041 10.1111/j.1728-4465.2007.00139.x

[25] Maman S, King E. Changes in HIV testing policies and the implications for women. J Midwifery Womens Health. 2008; 53(3):195–201. 10.1016/j.jmwh.2007.11.001 18455093

[26] Njeru MK, Blystad A, Shayo EH, Nyamongo IK, Fylkesnes K. Practicing provider-initiated HIV testing in high prevalence settings: consent concerns and missed preventive opportunities. BMC Health Serv Res. 2011; 11:87 10.1186/1472-6963-11-87 21507273 PMC3105945

[27] Fylkesnes K, Sandoy IF, Jurgensen M, Chipimo PJ, Mwangala S, Michelo C. Strong effects of home-based voluntary HIV counseling and testing on acceptance and equity: a cluster randomized trial in Zambia. Soc Sci Med. 2013; 86:9–16. 10.1016/j.socscimed.2013.02.036 23608089

[28] Jurgensen M, Sandoy IF, Michelo C, Fylkesnes K, Mwangala S, Blystad A. The Seven C’s of the high acceptability of home-based VCT: results from a mixed methods approach in Zambia. Soc Sci Med. 2013; 97:210–9. 10.1016/j.socscimed.2013.07.033 23972555

[29] Seeley J, Wagner U, Mulemwa J, Kengeya-Kayondo J, Mulder D. The development of a community-based HIV/AIDS counseling service in a rural area in Uganda. AIDS Care. 1991; 3(2):207–17. 1878404 10.1080/09540129108253064

[30] Sutton M, Anthony MN, Vila C, McLellan-Lemal E, Weidle PJ. HIV testing and HIV/AIDS treatment services in rural counties in 10 southern states: service provider perspectives. J Rural Health. 2010; 26(3):240–7. 10.1111/j.1748-0361.2010.00284.x 20633092

[31] Foster PH, Frazier E. Rural health issues in HIV/AIDS: views from two different windows. J Health Care Poor Underserved. 2008; 19(1):10–5. 10.1353/hpu.2008.0004 18263982

[32] Myers T, Worthington C, Haubrich DJ, Ryder K, Calzavara L. HIV testing and counseling: test provider’s experiences of best practices. AIDS Educ Prev. 2003; 15(4):309–19. 14516016 10.1521/aeap.15.5.309.23821

[33] Worthington C, Myers T. Desired elements of HIV testing services: test recipient perspectives. AIDS Patient Care STDS. 2002; 16(11):537–48. 12513902 10.1089/108729102761041092

[34] Beardsell S, Coyle A. A review of research on the nature and quality of HIV testing services: a proposal for process-based studies. Soc Sci Med. 1996; 42(5):733–43. 8685741 10.1016/0277-9536(95)00145-x

[35] Lie GT, Biswalo PM. Perceptions of the appropriate HIV/AIDS counselor in Arusha and Kilimanjaro regions of Tanzania: implications for hospital counseling. AIDS Care. 1994; 6(2):139–51. 8061074 10.1080/09540129408258625

[36] Grinstead OA, Van der Straten A, Voluntary HIV-1 Counseling and Testing Efficacy Study Group. Counselors’ perspectives on the experience of providing HIV counseling and testing in Kenya and Tanzania: the voluntary HIV-1 counseling and testing efficacy study. AIDS Care. 2000; 12(5):625–42. 11218548 10.1080/095401200750003806

[37] World Health Organization. Statement on HIV testing and counseling: WHO, UNAIDS re-affirm opposition to mandatory HIV testing Geneva: WHO; 2012.

[38] UNAIDS. UNAIDS report on the global AIDS epidemic Geneva: UNAIDS; 2013.

[39] WHO, UNAIDS, UNICEF. Global HIV/AIDS response: Epidemic update and health sector progress towards universal access Geneva: WHO; 2011.

[40] Topp SM, Chipukuma JM, Chiko MM, Wamulume CS, Bolton-Moore C, Reid SE. Opt-out provider-initiated HIV testing and counseling in primary care outpatient clinics in Zambia. Bull World Health Organ. 2011; 89(5):328–35. 10.2471/BLT.10.084442 21556300 PMC3089388

[41] Ministry of Health. Essential competencies: ART, PMTCT and CTC services Lusaka: Ministry of Health; 2006.

[42] Morris MB, Chapula BT, Chi BH, Mwango A, Chi HF, Mwanza J, et al Use of task-shifting to rapidly scale-up HIV treatment services: experiences from Lusaka, Zambia. BMC Health Serv Res. 2009; 9:5 10.1186/1472-6963-9-5 19134202 PMC2628658

[43] Ministry of Health. Zambia National Guidelines for HIV Counseling and Testing. Lusaka: Ministry of Health; 2006.

[44] Sanjana P, Torpey K, Schwarzwalder A, Simumba C, Kasonde P, Nyirenda L, et al Task-shifting HIV counseling and testing services in Zambia: the role of lay counselors. Hum Resour Health. 2009; 7:44 10.1186/1478-4491-7-44 19480710 PMC2692981

[45] Ministry of Health, National HIV/AIDS/STI/TB council. National HIV rapid testing training curriculum Lusaka: Ministry of Health; 2007.

[46] George P. University Teaching Hospital in Zambia: the strategic plan environment. Technical report no. 14. Bethesda, Maryland: Abt. associates Inc.; 1997.

[47] Graneheim UH, Lundman B. Qualitative content analysis in nursing research: concepts procedures and measures to achieve trustworthiness. Nurse Educ Today. 2004; 24(2):105–12. 14769454 10.1016/j.nedt.2003.10.001

[48] Weiser SD, Heisler M, Leiter K, Percy-de Korte F, Tlou S, DeMonner S, et al Routine HIV testing in Botswana: a population-based study on attitudes, practices and human rights concerns. PLOS Med. 2006; 3(7):1013–22. 10.1371/journal.pmed.0030261 PMC1502152 16834458

[49] Joo E, Carmack A, Garcia-Bunuel E, Kelly CJ. Implementation of guidelines for HIV counseling and voluntary HIV testing of pregnant women. Am J Public Health. 2000; 90(2):273–6. 10667191 10.2105/ajph.90.2.273 PMC1446152

[50] Chippindale S, French L. HIV counseling and the psychosocial management of patients with HIV or AIDS. BMJ. 2001; 322(7301):1533–5. 11420278 10.1136/bmj.322.7301.1533 PMC1120575

[51] Evans C, Ndirangu E. Implementing routine provider-initiated HIV testing in public health care facilities in Kenya: a qualitative descriptive study of nurses’ experiences. AIDS Care. 2011; 23(10):1291–7. 10.1080/09540121.2011.555751 21939406

[52] Gruskin S, Ahmed S, Ferguson L. Provider-initiated HIV testing and counseling in health facilities–what does this mean for the health and human rights of pregnant women? Dev World Bioeth. 2008; 8(1):23–32. 10.1111/j.1471-8847.2007.00222.x 18315722

[53] Wolpaw BJ, Mathews C, Chopra M, Hardie D, de Azevedo V, Jennings K, et al The failure of routine rapid HIV testing: a case study of improving low sensitivity in the field. BMC Health Serv Res. 2010; 10:73 10.1186/1472-6963-10-73 20307310 PMC2851712

[54] Chang D, Learmonth K, Dax EM. HIV testing in 2006: issues and methods. Expert Rev Anti Infect Ther. 2006; 4(4):565–82. 17009937 10.1586/14787210.4.4.565

[55] Clearview® Complete HIV 1/2 [package insert]. Medford, Newyork: Chembio Diagnostic systems; 2008.

[56] Gillet P, Mori M, Van den Ende J, Jacobs J. Buffer substitution in malaria rapid diagnostic tests causes false-positive results. Malar J. 2010; 9:215 10.1186/1475-2875-9-215 20650003 PMC3224932

[57] World Health Organization. Scaling up HIV testing and counseling services: a toolkit for programme managers Geneva: WHO; 2005.

[58] Mbilinyi D, Daniel ML, Lie GT. Health worker motivation in the context of HIV care and treatment challenges in Mbeya region, Tanzania: a qualitative study. BMC Health Serv Res. 2011; 11:266 10.1186/1472-6963-11-266 21992700 PMC3214153

[59] Kruse GR, Chapula BT, Ikeda S, Nkoma M, Quiterio N, Pankratz D, et al Burnout and use of HIV testing services among health care workers in Lusaka district, Zambia: a cross-sectional study. Hum Resour Health. 2009; 7:55 10.1186/1478-4491-7-55 19594917 PMC2714832

[60] Msisuka C, Nozaki I, Kakimoto K, Seko M, Ulaya MM. An evaluation of a refresher training intervention for HIV lay counselors in Chongwe District, Zambia. SAHARA J. 2011; 8(4):204–9. 10.1080/17290376.2011.9725005 23236962

[61] Ministry of Health. National Quality Assurance strategy for HIV counseling and testing Lusaka: Ministry of Health; 2007.

[62] Astles JR, Lipman HB, Schalla WO, Blumer SO, Fehd RJ, Smith C, et al Impact of quality control on accuracy in enzyme immunoassay testing for human immunodeficiency virus type 1 antibodies. Arch Pathol Lab Med. 1998; 122(8):700–7. 9701331

[63] Ministry of Health, Virology Laboratory. 1st proficiency testing trial preliminary draft report Lusaka: Virology Laboratory; 2009.

